# Molecular serotyping of clinical strains of *Haemophilus (Glaesserella) parasuis* brings new insights regarding Glässer’s disease outbreaks in Brazil

**DOI:** 10.7717/peerj.6817

**Published:** 2019-05-23

**Authors:** Julia Pires Espíndola, Natalia Balbinott, Letícia Trevisan Gressler, Gustavo Machado, Catia Silene Klein, Raquel Rebelatto, César Bernardo Gutiérrez Martín, Luiz Carlos Kreutz, Anthony Bernard Schryvers, Rafael Frandoloso

**Affiliations:** 1 Laboratory of Microbiology and Advanced Immunology, Faculty of Agronomy and Veterinary Medicine, University of Passo Fundo, Passo Fundo, Rio Grande do Sul, Brazil; 2 Department of Population Health and Pathobiology, College of Veterinary Medicine, North Carolina State University, Raleigh, NC, United States of America; 3 Embrapa Suínos e Aves, Concórdia, Santa Catarina, Brazil; 4 Section of Microbiology and Immunology, Department of Animal Health, Faculty of Veterinary, University of León, León, Castilla y León, Spain; 5 Department of Microbiology & Infectious Diseases, Faculty of Medicine, University of Calgary, Calgary, Alberta, Canada

**Keywords:** Disease mapping, *Haemophilus parasuis*, Typification, Serovars, Vaccines, Brazil

## Abstract

Glässer’s disease (GD) is an important infectious disease of swine caused by *Haemophilus (Glaesserella) parasuis*. Vaccination with inactivated whole cell vaccines is the major approach for prevention of *H. parasuis* infection worldwide, but the immunity induced is predominantly against the specific polysaccharide capsule. As a consequence, the available vaccines may not induce adequate protection against the field strains, when the capsules present in the vaccine strains are different from those in strains isolated from the farms. Therefore, it is crucial to map *H. parasuis* serovars associated with regional outbreaks so that appropriate bacterin vaccines can be developed and distributed for prevention of infection. In this study, 459 *H. parasuis* field strains isolated from different Glässer’s disease outbreaks that occurred in 10 different Brazilian States were analyzed for serotype using PCR-based approaches. Surprisingly, non-typeable (NT) strains were the second most prevalent group of field strains and along with serovars 4, 5 and 1 comprised more than 70% of the isolates. A PCR-based approach designed to amplify the entire polysaccharide capsule locus revealed 9 different band patterns in the NT strains, and 75% of the NT strains belonged to three clusters, suggesting that a number of new serovars are responsible for a substantial proportion of disease. These results indicate that commercially available vaccines in Brazil do not cover the most prevalent *H. parasuis* serovars associated with GD.

## Introduction

*Haemophilus (Glaesserella) parasuis* is a pleomorphic, NAD-dependent, Gram-negative bacterium of the *Pasteurellaceae* family (Biberstein & White, 1969). *H. parasuis* normally resides in the upper respiratory tract of pigs but can also cause a systemic infection named Glässer’s disease (GD), which is characterized by pneumonia, fibrinous polyserositis, polyarthritis and meningitis (Oliveira, Galina & Pijoan, 2001). Since a polysaccharide capsule is invariably present in GD disease isolates, it has formed the basis of a serovar (SV) classification scheme used for *H. parasuis* (Kielstein & Rapp-Gabrielson, 1992). Certain *H. parasuis* serovars have been associated with high mortality rates in swine herds worldwide, resulting in substantial production losses and increased costs mainly due to the use of antibiotics, especially when piglets from different sources are mixed (Møller et al., 1993; Nedbalcova et al., 2006).

An immunodiffusion assay has been used to classify *H. parasuis* into 15 serovars (SVs 1 to 15) amongst which SVs 1, 5, 10, 12, 13 and 14 were considered highly virulent; SVs 2, 4, 8 and 15 moderately virulent; and SVs 3, 6, 7, 9 and 11 were characterized as having low virulence potential (Kielstein & Rapp-Gabrielson, 1992). A recent study demonstrated that the *H. parasuis* 174 strain, the reference strain for SV7, was virulent, suggesting that some caution be exercised in associating virulence to capsular types (Guizzo et al., 2018). There have been reports of non-typeable strains responsible for disease outbreaks suggesting that there are other types of polysaccharide capsule in *H. parasuis* strains or that strains lacking capsular are capable of causing disease. Commercial vaccines are major tools to control GD; however, most globally available vaccines are based on whole inactivated cells from one or two SVs of *H. parasuis* (Porcilis Glässer, Intervet (SV5), Rhinanvac cerdos, Syva (SV5), Suvaxyn® Respifend® MH/HPS, Zoetis (SV4 and SV5), Hiprasuis® Glässer, Hipra (SV1 and SV 6), Ingelvac® HP-1, Boehringer Ingelheim (strain Z-1517, SV not available)) or an attenuated whole cell vaccine (Parasail®, Newport Laboratories, SV5). In addition, not all vaccines are available in Brazil and those available do not cover the wide SV diversity reported to be present. It is likely that this situation applies to many other countries worldwide.

The protective efficacy of the inactivated whole cell vaccines (bacterins) is largely against isolates belonging to the same SV with limited cross-protection (Bak & Riising, 2002; Smart & Miniats, 1989; Takahashi et al., 2001). This suggests that vaccine induced antibodies are predominantly against capsule and the factors that influence the induction of cross-protective antibodies have not been determined. Thus, the continued report of outbreaks of GD due to failure of bacterin vaccines are a major concern to researchers and swine producers.

In order to rationally design bacterin vaccines, it is necessary to have a clear picture of the most prevalent SVs in each pig production region. SVs 4 and 5 are the most prevalent serovars in several countries in which pig production is a major economic activity (Angen, Svensmark & Mittal, 2004; Blackall, Rapp-Gabrielson & Hampson, 1996; Rapp-Gabrielson & Gabrielson, 1992; Rubies et al., 1999; Zhang et al., 2012), thus the development of the commercially available vaccines was based on these SVs. Studies evaluating the serovar prevalence are limited in Brazil, the fourth largest swine producer in the world (ABPA, 2015), where there has been a steady increase in GD outbreaks that may be correlated with vaccine failure. This suggests a need for typing of *H. parasuis* clinical isolates in Brazilian herds to facilitate the design of regional, more effective bacterins containing antigens from the locally most prevalent SVs.

To address this issue, we performed typing of 459 *H. parasuis* strains isolated from 10 different Brazilian States in which pig production is an important economic activity. In addition, we describe a new set of primers designed to amplify the capsular polysaccharide locus of non-typeable strains useful to assess the strain diversity into this group. The results of this study show: (a) the identification, classification and map distribution of *H. parasuis* SVs related to GD outbreaks in Brazil; (b) circulation at least nine different non-typeable strains based on the PCR patterns from the capsular loci; and (c) most GD outbreaks (71.9%) were caused by SVs not included in commercial vaccines available in Brazil, underlying the necessity of revising the antigen composition of these vaccines.

## Material & methods

### Clinical isolates

*H. parasuis* field strains (*n* = 459) used in this study were isolated from 30 to 70 day old pigs involved in different clinical outbreaks of GD. One hundred forty-five of these strains were obtained from the Laboratory of Microbiology and Advanced Immunology of the University of Passo Fundo from 2012 to 2016. One hundred and two were isolated in the Bacteriology section of Embrapa Swine and Poultry Research Center from 1987 to 2013 and 212 were isolated by the “Instituto de Pesquisas Veterinárias Especializadas Ltda” (IPEVE, Brazil) from 2012 to 2016. All isolates were recovered from either the lungs, heart, brain, joints or trachea of pigs with clinical signs (high fever, arthritis, tremors, incoordination, lateral recumbency and depression) and post mortem lesions (polyserositis) compatible with GD. Each strain represents one single animal studied. All strains were stored at −80 °C. Along with herd data and the individual medical history, for each isolate there is information on clinical signs, date, city, state and sampling tissue (except for 16 isolates where the tissue source was missing). The *H. parasuis* reference strains N°4, SW140, SW114, SW124, Nagasaki, 131, 174, C5, D74, H555, H465, H425, 84-17975, 84-22113 and 84-15995 were used to represent all the 15 known serovars (Kielstein & Rapp-Gabrielson, 1992).

### DNA extraction

For the molecular typing of *H. parasuis* strains, the bacteria were grown in chocolate agar and incubated for 24–36 h at 37 °C in an atmosphere containing 5% CO_2_. A loop of pure culture was suspended in 200 µl of ultrapure water (Sigma-Aldrich, USA), which was heated to 95 °C for 10 min and centrifuged at 13,000× g for 10 min. The supernatant containing DNA was quantified, transferred to a new tube and used in the PCR reactions.

To optimize PCR amplification with primers designed to amplify the capsular locus using appropriate enzymes (see below) total genomic DNA (gDNA) from the 15 reference strains and 70 non-typeable *H. parasuis* field strains were extracted using Wizard® Genomic DNA Purification Kit (Promega, Madison, WI, USA) following the manufacturer’s instruction. The total gDNA was quantified by spectrophotometry (NanoDrop; Thermo Scientific, Waltham, MA, USA), diluted to 20 ng/µl and stored at −20 °C until use.

### Serotyping

The clinical isolates were first typed by a multiplex PCR (mPCR) method which is capable of differentiating 13 of the 15 known serovars (Howell et al., 2015). Gel electrophoresis of the PCR products was performed in a 2.0% agarose gel in Tris-acetate-EDTA (TAE) at 120 volts for 90 min. The DNA products were stained with GelRed™ (Biotium, Fremont, CA, USA) and the fragment sizes were compared to a 50-bp DNA ladder (Ludwig, Brazil). The gDNA from the 15 *H. parasuis* reference strains were used to validate the mPCR method. SVs 5 and 12 could not be differentiated by the mPCR approach (Howell et al., 2015), thus these samples were analyzed by a conventional PCR using primers specific for *H. parasuis* SV12 (SV12.F ATGGCTCACGATCCGAAAG and SV12.R ATTTCCCTTTCCTAAACGC) (Jia et al., 2017).

### Capsular polysaccharide locus amplification

The full-length nucleotide sequences of the genes *funA* and *wza* from the 15 reference *H. parasuis* strains sequenced by Howell et al. (2013) were obtained from GenBank, aligned and compared using Gene Inspector® software (Textco BioSoftware, West Lebanon, NH, USA) for primer design. Two common sequences were identified and used to design the forward primer on the *funA* gene (5′-GTGCTAGAAAACGGACGCTACATAG-3′, labeled as funAU) and the reverse primer on the *wza* gene (5′-CCATGACGAGTAATAGTCACATTATGCC-3′, labeled as wzaU). PCR reactions were prepared with following components, 10 µL of 5× Phusion HF Buffer, 300 µM of each deoxynucleotide triphosphate (dNTP, Invitrogen, USA), 0.5 µM of each primer, 0.01 U Phusion DNA Polymerase (Thermo Scientific, Waltham, MA, USA) and 100 ng of gDNA in a final volume of 50 µL. The cycling conditions were 30 s at 98 °C, followed by 35 cycles of 10 s at 98 °C, 30 s at 57 °C and 6 min at 72 °C, then a final extension at 72 °C for 10 min. Gel electrophoresis of the PCR products was performed in a 0.6% agarose gel in TAE at 90 volts for 60 min. The DNA products were stained with GelRed™ (Biotium, Fremont, CA, USA) and the fragment sizes were compared to a 1 kb DNA ladder (O’GeneRuler 1 kb Plus DNA Ladder; Thermo Scientific, Waltham, MA, USA).

### Data analyses

Data from all samples were analyzed as descriptive statistics. Each SV was assigned as belonging to a high or a moderate virulence group according to the Kielstein & Rapp-Gabrielson (1992) scheme. Geographical visualization was performed using ArcGIS 10.4 (Esri ArcMap; Esri, Redlands, CA, USA).

**Table 1:** Organ of isolation and serotyping distribution of *H. parasuis* field isolates recovered from pigs suffering Glässer’s disease in Brazil.

	**Site of Isolation**	**Serovars**^a^									**Total (%)**
		**NT**^b^	**1**	**2**	**4**	**5**	**12**	**13**	**14**	**15**	
Systemic	Brain	5	1		1	5			4		16 (3.5%)
	Heart	3	5	2	11	11		1	4	1	38 (8.3%)
	Joints		1		2				3		6 (1.3%)
	Peritoneum	1			1				2		4 (0.9%)
	Pleural cavity				1	2	1		2	1	7 (1.5%)
	Spleen					1					1 (0.2%)
Respiratory	Lungs	50	40	3	98	45	33	2	35	13	319 (69.5%)
	Nasal cavity	10					1		1	4	16 (3.5%)
	Trachea	8	8		6	3	4		4	3	36 (7.8%)
	Not determined	4	5		2	2			3		16 (3.5%)
	Total	81 (17.6%)	60 (13.1%)	5 (1.1%)	122 (26.6%)	69 (15%)	39 (8.5%)	3 (0.7%)	58 (12.6%)	22 (4.8%)	459 (100.0%)

**Notes.**

a Serotyping was performed by means of mPCR.

b NT, nontypeable isolates.

## Results

### *H. parasuis* typing and site of isolation

The strains obtained from the indicated tissue source (Table 1) were serotyped as described in the ‘Materials and Methods’ section. *H. parasuis* SV4 was the most frequent isolate found in this study (26.6%) followed by NT isolates (17.6%), and those belonging to SV5 (15%) and SV1 (13.1%). Initially, 108 (23.5%) of the isolates were classified as SV5/12 by multiplex PCR (mPCR) and were then analyzed by uniplex PCR (specific primers for SV12) to obtain the final classification as SV5 or SV12.

**Figure 1:**
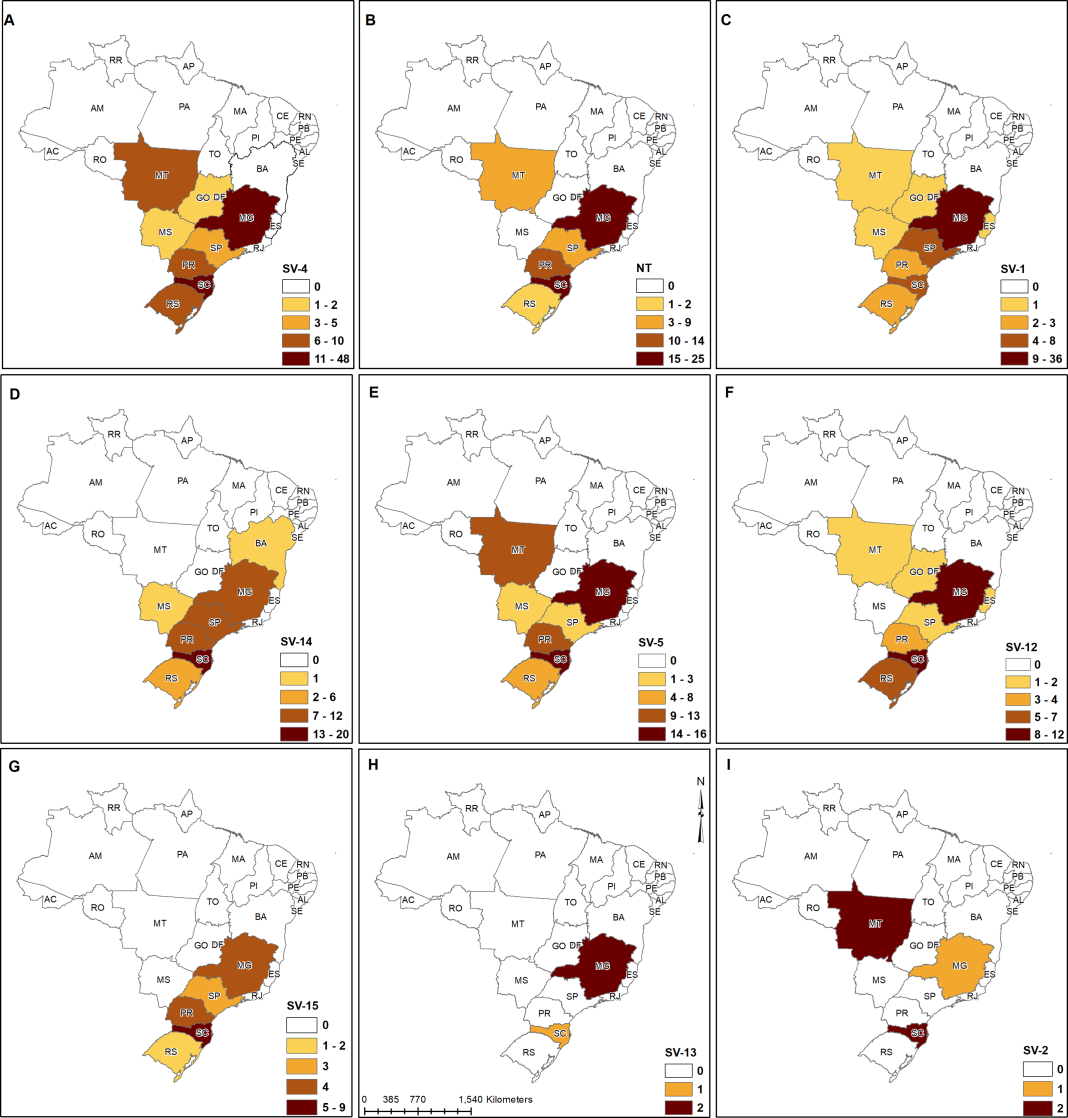
Geographic distribution of the most prevalent *H. parasuis* serovars for each of the ten Brazilian states. (A) serovar 4, (B) non-typeable *H. parasuis*, (C) serovar 1, (D) serovar 14, (E) serovar 5, (F) serovar 12, (G) serovar 15, (H) serovar 13, (I) serovar 2.

### Geographical distribution of clinical isolates

**Figure 2:**
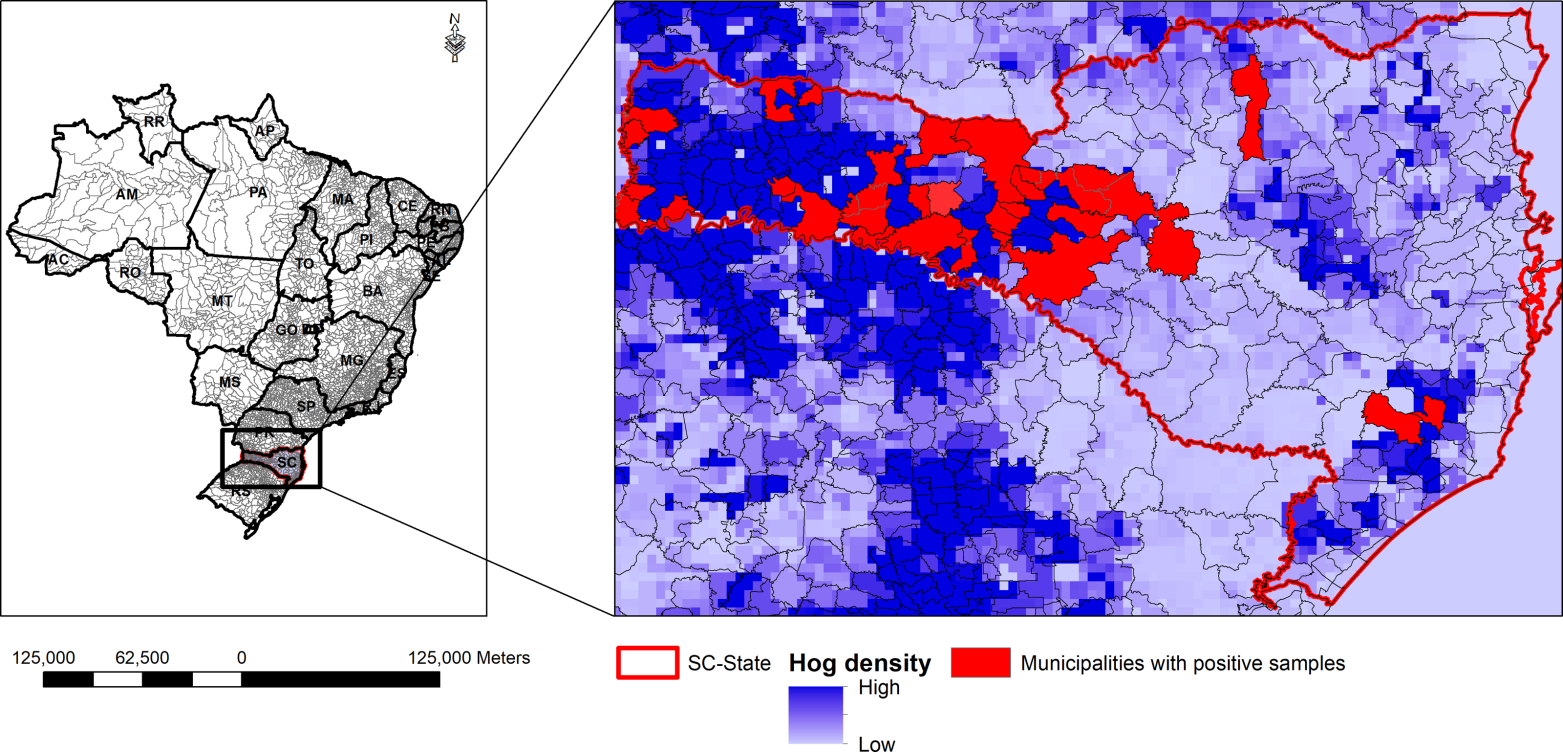
Density map of the municipalities with Glässer’s disease outbreaks in Santa Catarina (SC) States. The red fill area represents GD outbreaks diagnosed in the different counties of SC, which are ranked as the first in pig production of Brazil.

Clinical isolates were obtained from GD outbreaks from ten Brazilian states: Bahia (BA), Espírito Santo (ES), Goiás (GO), Mato Grosso (MT), Mato Grosso do Sul (MS), Minas Gerais (MG), Paraná (PR), Rio Grande do Sul (RS), Santa Catarina (SC) and São Paulo (SP) (Fig. 1). The majority were from MG (33.8%) and SC (27.6%), and isolates belonging to almost all SVs were found in these two states. The presence of SV2 was detected in SC, MG and MT states, while the SV13 was limited to SC and MG states. Since SC is responsible for producing 26.7% of the total pigs raised in Brazil (ABPA, 2015), we generated a density map of pig production and highlighted the sites from which isolates were obtained to illustrate that the sampling was reasonably representative of pig infections in the state of Santa Catarina (Fig. 2).

### Temporal distribution of *H. parasuis* field isolates

**Figure 3:**
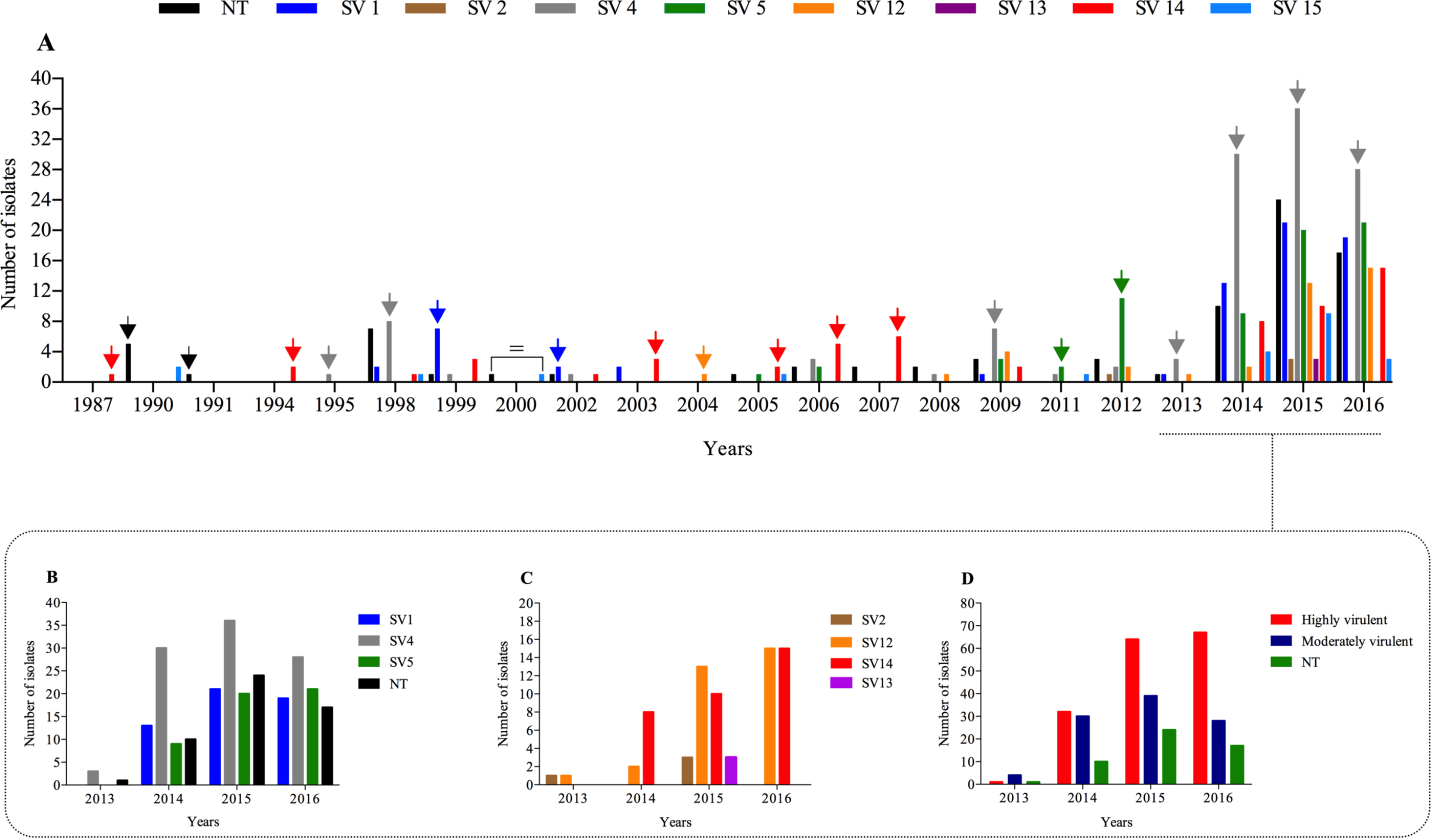
Distribution over the years of count number of *H. parasuis* field isolates. The *y*-axis represents the count observations and *x*-axis the correspondent isolation year. (A) Number and serovar of strains isolated over the 22 years period. (B) Serovars most prevalent isolated from 2013 to 2016, which represent 69.9% of field strains analyzed. (C) Serovars less prevalent isolated from 2013 to 2016. (D) Number of isolates grouped according to virulence class (serovars) or non-typeable (NT).

The distribution of the serovar of strains isolated over the 22-year period is illustrated in Fig. 3A. The most prevalent *H. parasuis* SVs from 2013 to 2016 is shown in Fig. 3B and the less prevalent SVs are shown in Fig. 3C. SV4 was the most frequently recovered from 2013 to 2016 (97 isolates) followed by SV1 (53 isolates), NT (52 isolates) and SV5 (50 isolates). The recovery of clinical isolates belonging to SV13 first occurred in 2015 and the frequency of SV14 has increased notably from 2014 (Fig. 3A). When the SVs were grouped according to their proposed virulence, a rise of the highly virulent isolates was observed from 2013 onwards (Fig. 3D).

### *H. parasuis* non-typeable strains: capsule locus analysis

**Figure 4:**
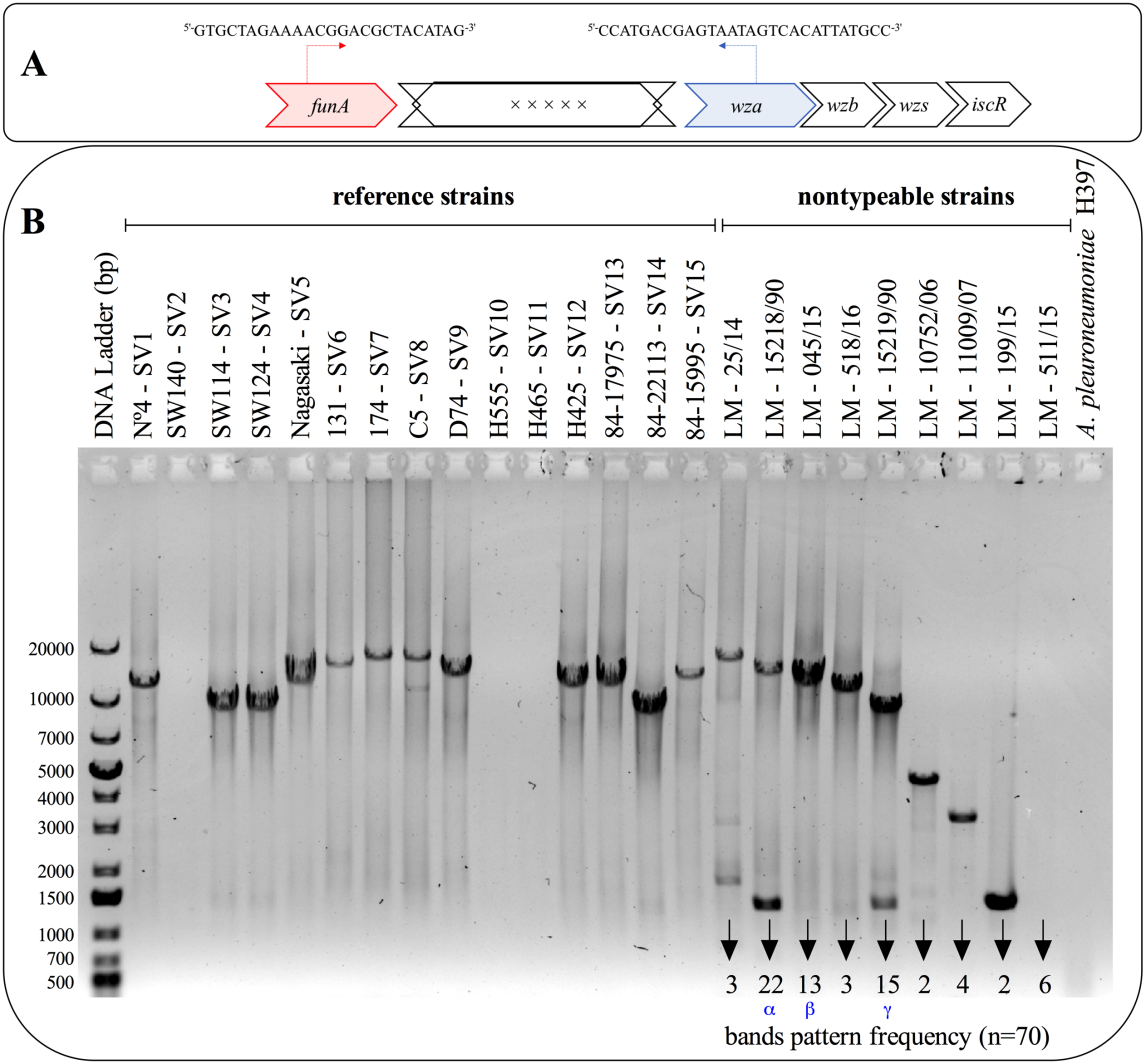
Molecular capsular polysaccharide locus analysis. (A) Illustrative scheme of the localization of funAU and wzaU primers into the locus. (B) Band patterns of the partial capsule polysaccharide locus amplification for all 15 reference serovars of *H. parasuis* and for 70 non-typeable Brazilian strains.

In an attempt to determine whether the non-typeable *H. parasuis* strains have a locus responsible for the synthesis of capsular polysaccharides we designed primers to conserved regions in the *funA* and *wza* genes to amplify the entire region encoding the specific genes for synthesis of the polysaccharide capsule (Fig. 4A). The rationale for this approach is that strains lacking an extracellular polysaccharide capsule would be expected to have a small PCR product whereas strains with an extracellular polysaccharide capsule would have a large PCR product. PCR amplification using genomic DNA from the 15 reference strains of *H. parasuis* resulted in large PCR products in 12 of the 15 reference strains. Clearly the failure to obtain large PCR products for three of the serotype strains, SW140 (SV2), H555 (SV10) and H465 (SV11), is not due to an absence of the locus but illustrate the limitations of the approach with a single set of conserved primers. When this primer set was used on the non-typeable strains, the locus of 64 of the 70 strains were amplified and the capsule size pattern amongst the strains allowed us to classify them into nine patterns (Fig. 4B). Three dominant patterns were observed, labeled by *α*, *β* and *γ*, representing 31.4%, 18.6% and 21.4% of the total non-typeable strains analyzed, respectively.

## Discussion

Vaccination with inactivated whole cell vaccines is widely used worldwide and represents the major tool in reducing economic losses associated with Glässer’s disease. However, the inherent heterogeneity in serovar type observed in *H. parasuis* isolates hamper the development of effective immunity to prevent the infection caused in pigs by different SVs than those included in the vaccines marketed against this disease. Currently, Brazil has three commercial vaccines that are composed of SV5 (Porcilis Glässer, Intervet) (Segers, Witvliet & Hensen, 2009), mixtures of SVs 1 and 6 (Hiprasuis Glässer, HIPRA), and one vaccine for which the serovar is not provided (*H. parasuis* strain Z-1517, Ingelvac® HP-1, Boehringer-Ingelheim) (Swart et al., 2014). The selection of these serovars to formulate these global vaccines were based on the results obtained from epidemiological studies carried out in other countries.

For our study, a total of 459 clinical strains of *H. parasuis* were isolated from pigs representing different farms with Glässer’s disease outbreaks. The strains were collected over the last 22 years from 10 different Brazilian states, which covers all regions with industrial pig production. The areas sampled (Figs. 1 & 2) were primarily from the major pig producing states (RS, SC, PR and MG; 88% of strains) thus is a reasonable representation of the serovars of *H. parasuis* responsible for Glässer’s disease in Brazil.

Molecular typing is an excellent alternative test compared to regular serotyping (gel immunodiffusion, Kielstein and Rapp-Gabrielson scheme), which is very cumbersome to perform because of the necessity of producing specific anti sera. Using a combination of a multiplex PCR assay (Howell et al., 2015) and a specific PCR reaction for the *H. parasuis* SV12, 8 different SVs were identified. A significant proportion (17.6%) of the isolates were NT in ten Brazilian States, showing the diversity and widespread dissemination of *H. parasuis* across the states. SVs 4 and 5 represented 50.5% of the typed isolates, a finding which does not vary from previous reports, in which SV4 was also the most prevalent, followed by SVs 5, 14 and 13 (Castilla et al., 2012; Macedo et al., 2011; Moreno et al., 2011). Notably, SV1 was responsible for 13.1% of GD outbreaks observed in this study, mainly in MG (36 cases, Fig. 1), underlying the importance of the *H. parasuis* typing since a commercial vaccine formulated with this serovar is currently available in the country.

The prevalence of clinical strains belonging to the SV4 is a problem for Brazil since the only global commercial vaccine (Suvaxyn® Respifend® MH/HPS) that could potentially protect against this serovar is not available in Brazil. Since 17 of the 122 farms with Glässer’s disease outbreaks caused by SV4 were using one of the three available commercial vaccines in Brazil during the outbreaks (supplementary information), it is reasonable to assume that the absence of protection can be due to a limited cross protection against this serovar.

Assuming that the protection with inactivated bacterial vaccines is serovar specific (Rapp-Gabrielson et al., 1997; Takahashi et al., 2001), the currently available vaccines could have potentially protected a maximum of 28.1% of the GD outbreaks reported in this study, those caused by SVs 1 and 5. If one presumes that the cross-reactivity observed in typing the SV5 and SV12 strains translates into cross-protection, as described by Bak & Riising (2002), the estimated level of protection could reach 36.6%. These results highlight just how susceptible the majority of the vaccinated pigs are to *H. parasuis* infection caused by SVs 2, 4, 13, 14, 15 and NT, which is of great concern for the fourth largest pig producer worldwide.

When commercial vaccines fail, the most appropriate short-term solution to the problem is the use of autogenous vaccines, which is more effective when prepared from isolates collected from systemic sites of *H. parasuis* infection, like meninges, pericardium and joints (Oliveira & Pijoan, 2004; Smart, Miniats & MacInnes, 1988). In spite of the fact that a well-established recommendation is available for the correct collection of samples from a pig suffering Glässer’s disease, our study (Table 1) illustrates that the tissues most frequently sent for diagnosis in Brazil are from the respiratory tract (lung 69.5%, trachea 7.8% or nasal cavity 3.5%). This fact can compromise the rational design and protecting power of autogenous vaccine formulated with field isolates.

It is also important to perform molecular analyses of the disease-causing strains in parallel to autogenous vaccine preparation, to determine whether autogenous vaccine failure is a result of mis-match of strains, or to guide the development of commercial vaccines that will be effective in the region. Although a number of molecular typing methods are available, ERIC-PCR (Rafiee et al., 2000) is a discriminatory technique suitable for maintaining a database of strains from the same herd over time, and to verify the necessity of strain inclusion in the autogenous vaccine due to new outbreaks. Although autogenous vaccines are the most logical short-term solution, and typing methods can guide the development of commercial vaccines based on the appropriate capsular serotypes, it is unlikely to completely prevent disease, particularly since our study shows that one cannot predict or prevent the appearance of new serovars capable of causing infection. Thus, the most effective long-term solution is development of more cross-protective protein-based vaccines such as one targeting surface transferrin receptors that is predicted to completely prevent disease by *H. parasuis* (Barasuol et al., 2017; Curran et al., 2015; Guizzo et al., 2018), and could potentially eliminate several porcine pathogens from pig barns if it is effective at preventing natural colonization.

Non-typeable *H. parasuis* strains are associated with GD outbreaks globally but there is limited information about the molecular (Ma et al., 2016) or serological characterization of these strains. In our study, we designed a set of primers (funAU and wzaU, Fig. 4A) that allowed us to classify 70 non-typeable strains by nine different patterns, based on the PCR product size or pattern (Fig. 4B). Our analysis also allowed us to group 75% of the NT strains into 3 major clusters (*α*, *β* and *γ*), which could even be used as a preliminary identification of NT strains that will be used for the production of autogenous vaccine. This PCR approach may be a first step in characterizing non-typeable *H. parasuis* strains which could be complemented by serological characterization of new serovars of this microorganism, although it may be preferable to use a more discriminatory technique such ERIC-PCR (Rafiee et al., 2000). Our results demonstrate that there are strains with uncharacterized polysaccharide capsules capable of causing GD which adds to the complexity of achieving effective protection against *H. parasuis* through the use of classical vaccines.

Finally, the inability to amplify the capsule locus of three reference strains (SW140, H555 and H465) was not expected since, (i) the *in-silico* analysis showed a 100% of homology between the primer sequences and the nucleotide sequence of the three genomic DNA preparations, and (ii) the expected size of this region was less than 20 kb in the range of capsule loci that were successfully amplified.

## Conclusion

In conclusion, this study reveals that the commercially available vaccines in Brazil do not include the most prevalent SVs isolated from pigs suffering GD and that a substantial proportion of disease was caused by non-typeable (NT) strains that likely could represent a number of new serovars. To overcome this problem in the short-term, commercial formulations used in other countries, which incorporate SV4 in its composition, might benefit the Brazilian pig production, providing that the SV composition does not change. The relatively high prevalence of NT isolates that were obtained from most of the Brazilian states suggest that a number of new bacterins are needed or that alternate approaches for more cross-protective vaccines need to be considered.

##  Supplemental Information

10.7717/peerj.6817/supp-1 Supplemental Information 1 Raw data used to prepare Figs. 1–4 and Table 1 The record of each strain including serotyping, geographic localization, year of sampling and vaccination history of several farms before outbreaks of GD are described.

## Additional Information and Declarations

### Data Availability

The following information was supplied regarding data availability: The raw data are available in the Supplemental File. The record of each strain including serotyping, geographic localization and year of sampling are included. Additionally, information about the vaccination of several farms before GD outbreak is described.
